# Assessment of Saliva Oxidative Stress Biomarkers and Gingival Health Status in a Sample of High-Functioning Autistic Children in Erbil City

**DOI:** 10.7759/cureus.73717

**Published:** 2024-11-15

**Authors:** Shno S Mahmood, Ali F Alzubaidee, Vian M Hussein

**Affiliations:** 1 Special Care Dentistry, Helena Center for Special Needs, Erbil, IRQ; 2 Oral Medicine, Kurdistan Higher Council of Medical Specialties, Erbil, IRQ; 3 Dental Public Health, Kurdistan Higher Council of Medical Specialties, Erbil, IRQ

**Keywords:** autistic children, biomarkers, erbil city, gingival health, saliva oxidative stress

## Abstract

Introduction

Autism spectrum disorder (ASD) is a complicated disorder that affects communication, social interaction, and behavior. Several investigations have documented increased oxidative stress and damage in individuals with ASD compared with neurotypical controls. Saliva can be used as a non-invasive technique to assess oxidative stress biomarkers. This study aimed to explore the association between oxidative stress and oral health in a sample of high-functioning children with autism in Erbil City.

Methods

We conducted a case-control study with 96 participants aged 5-12 years, which included 48 children with ASD and 48 healthy controls. Stimulated saliva samples were collected and centrifuged. Oxidative stress biomarkers (malondialdehyde [MDA], glutathione [GSH], and uric acid) and gingival/plaque indices were also measured. Data were analyzed using SPSS, with significance set at p≤0.05.

Results

The data showed no significant differences between the ASD and control groups in the gingival index, salivary malondialdehyde, glutathione, or uric acid levels. However, the control group had a significantly higher mean plaque index than the ASD group (P = 0.003).

Conclusion

Sample size and confounding variables may influence the absence of significant differences in gingival index and salivary oxidative stress biomarkers between groups. The higher plaque index in controls aligns with plaque gingivitis. Age differences could impact oral health interpretations. Further research is needed to understand significant factors and the clinical significance of these findings in pediatric ASD populations. Most autistic children were from educated families and had good oral hygiene in addition to special care by the staff of the center.

## Introduction

Autism spectrum disorder (ASD) is a complex condition that influences a person's ability to communicate, relate to others, and regulate behavior. People with ASD often have unique interests, preferences, and ways to interact with the world. They may experience challenges in communication, social interactions, and sensory processing [1]. They also have many strengths and abilities, such as attention to detail [2], creativity, and a deep understanding of their interests [1,3]. The international incidence of ASD has significantly increased in recent decades, from approximately 0.05% during the 1960s to approximately 1-2% [4]. This promotion is probably due to improved understanding and changes in diagnostic criteria, although some functions of the environmental aspects cannot be ruled out [5].

The observed advancements in ASD understanding, attributed to improved diagnostic standards, are significant. However, it is crucial to also consider the potential impact of environmental factors [2]. Oxidative stress plays a critical role in both ASD and gingival health. Studies show elevated oxidative stress in ASD, marked by increased malondialdehyde (MDA) and altered antioxidant defenses like glutathione (GSH). It is reassuring that several analyses, including those by Rose et al. [6], Rossignol and Frye [7], and Frustaci et al. [8], have proven increased oxidative stress and harm in individuals with ASD compared to neurotypical counterparts. These biomarkers are significant for assessing oxidative damage, where MDA indicates lipid peroxidation and GSH serves as a primary antioxidant. In gingival disease, similar oxidative mechanisms contribute to inflammation, establishing relevance to both ASD and gingival health assessments. Numerous studies have shown that individuals with ASD experience heightened oxidative stress and injury compared to those without ASD, who exhibit normal neurological development [9,10].

Saliva is a representative indicator of the typical oral milieu and may serve as a valuable, non-intrusive approach for considering the state of oxidative stress [11]. Few studies have examined salivary oxidative stress biomarkers in children with ASD. Interestingly, the higher the level of this marker, the more severe the autism symptoms in children. This suggests that oxidative stress, a type of cellular damage caused by an imbalance of free radicals, may play a role in the development or severity of autism [12,13].

Aside from the evidence of systemic oxidative stress, there are indications that oxidative stress could impact oral health in ASD patients. Children with ASD often grapple with developmental, behavioral, and sensory issues that affect their ability to engage in effective oral hygiene practices [14]. They frequently exhibit restricted and selective eating patterns, leading to inadequate nutrient intake and compromised oral health care [15]. Poor oral hygiene and diet are likely contributing factors to the high rates of gingivitis and dental caries observed in patients with ASD [16,17]. Oxidative injury to oral tissues could be an additional contributing factor, highlighting the importance of understanding the relationship between oxidative stress and oral health in ASD patients.

The documented high prevalence of gingivitis and dental caries in individuals with ASD is likely impacted by inadequate oral hygiene practices and dietary habits [18]. Oxidative damage to oral tissues may serve as an additional contributing factor, and children with autism have poorer dental hygiene and gingiva health than children without autism. Not only were their gingiva less healthy, but they also had higher levels of oxidative stress markers in their saliva. This is an exciting finding, as gingiva inflammation and immune responses can lead to even more oxidative stress, which is similar to a vicious cycle. It resembles a never-ending loop in dental drama [19]. Few studies have described gingival health and salivary parameters in children with autism, as reported by Hafez et al. [20]. The study showed that children with autism seem to have higher gingival inflammation, poor oral hygiene, and a slightly lower salivary pH than the healthy control group.

Gingivitis is an inflammation of the gingival tissues induced by plaque buildup on the surface of the teeth. Plaque, the bacteria on the tooth surface, irritates the gingival tissues and causes bleeding and swelling. Effective oral hygiene, including frequent brushing and flossing, is essential to prevent and manage gingiva disease [21].

This study aimed to explore the relationship between oxidative stress and oral health in a sample of high-functioning autistic children in Erbil City by estimating salivary oxidative stress indicators and assessing gingival health status. This analysis aims to clarify potential linkages between oxidative stress and oral health in the population of interest.

## Materials and methods

Study design and setting

This study was designed as a case-control study, and healthy and ASD children aged 5-12 years were tested for salivary oxidative stress biomarkers and gingival health status. The study was conducted at the Helena Medical Center and Khanzad Dental Center in the Erbil-Kurdistan region of Iraq from the beginning of January 2023 until the end of January 2024. This study was undertaken to assess the prevalence of gingival disease among ASD patients; the researchers tested oxidative stress in ASD patients and checked the association between it and plaque and gingival indices.

Sampling

A power analysis was conducted, targeting a medium effect size (Cohen’s d = 0.5) with 0.8 power and an alpha of 0.05. Accordingly, 90 participants were determined to be sufficient for statistical reliability.

Method and data collection

The study was conducted with a sample size of 90 subjects categorized into two groups: the autistic group comprising 45 children and 45 healthy children categorized as a control group who attended both medical centers. Stimulated saliva was collected using a suction tube in the morning from 8 to 11 am [21] and then centrifuged at 1000 rpm for 10 minutes, after which the saliva was collected for one hour to eliminate debris and cellular matter. The samples were stored at −20 °C in a polyethylene tube until assayed in a freezer. We compared oxidative stress levels in ASD and the control group by introducing parents through direct interviews to a questionnaire provided with modified gingival and plaque indices and oxidative stress biomarkers in addition to sociodemographic data of participants, namely age, sex, residence, and socioeconomic status.

Biomarkers MDA and GSH were assessed using established biochemical assays. MDA was quantified using TBARS, a reliable marker of lipid peroxidation, and GSH via enzymatic analysis. Uric acid was analyzed with a validated colorimetric method. Calibration and reliability checks ensured accuracy across assays.

Data management and statistical analysis

The data were recorded on a specially designed questionnaire, collected and entered into the computer via Microsoft Excel worksheet (Microsoft® Corp., Redmond, WA), and then analyzed using the appropriate data system, Statistical Package for Social Sciences (SPSS, IBM Corp., Armonk, NY) version 28. The results were compared between groups with different variables (age, sex, residence, and SES), with a statistical significance level of ≤ 0.05. The results were presented as rates, ratios, frequencies, and percentages in tables and figures and analyzed using t-tests and chi-square tests.

Inclusion criteria

This study included both the ASD group and control (healthy) participants as well as high-functioning (level 1) ASD patients.

Exclusion criteria

Patients with systemic diseases of ASD and the control group from both genders, parents of respondents who did not give consent, and low-functioning (level 3) ASD patients who were not cooperative were excluded from the study.

Ethical consideration

This study received ethical approval from the Kurdistan Higher Council of Medical Specialties Ethics Committee (Approval No. 2275-08/12/2022). Written consent was obtained from all participants' parents, ensuring adherence to ethical standards. Confidentiality and anonymity of data were ensured.

## Results

**Figure 1:**
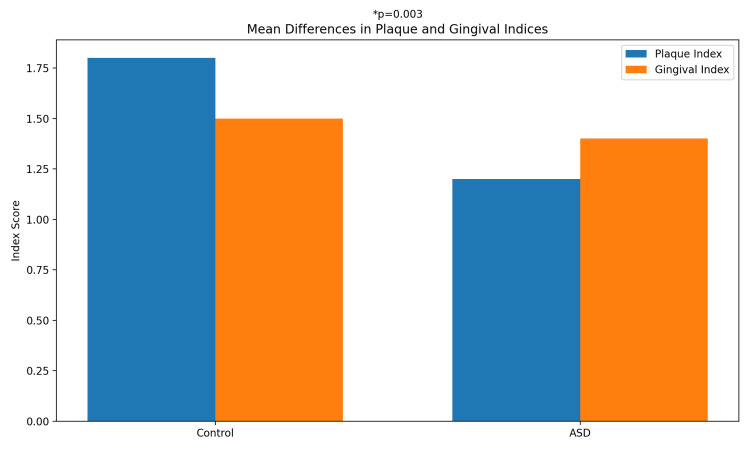
Mean differences in plaque and gingival indices.

Participant age distribution indicated that the mean age of participants was 8.38 ± 2.19 years. Assessment of the gingival and plaque index showed that the mean gingival index was 0.06 ± 0.166 scale, and the mean plaque index of patients was 0.31 ± 0.39 scale. The mean salivary malondialdehyde was 143.48 ± 204.28 μM/L, the mean salivary glutathione was 13.66 ± 18.37 μM/L, and finally, the mean uric acid was 25.95 ± 13.37 mg/dL (Table 1 and Figure 1).

**Table 1:** Mean and standard deviation of age, gingival, plaque indices, salivary malondialdehyde, glutathione, and uric acid of the whole sample size.

Variables	N	Range	Minimum	Maximum	Mean	Std. deviation
Age (years)	90	10	2	12	8.38	2.19
Gingival index (scale)	90	0.83	0	0.83	0.06	0.166
Plaque index (scale)	90	1.8	0	1.8	0.31	0.39
Salivary malondialdehyde (μM/L)	90	1311	19	1330	143.48	204.28
Salivary glutathione (μM/L)	90	115.8	1.4	117.2	13.66	18.37
Uric acid (mg/dL)	90	61.6	6.1	67.7	25.95	13.37

**Figure 2:**
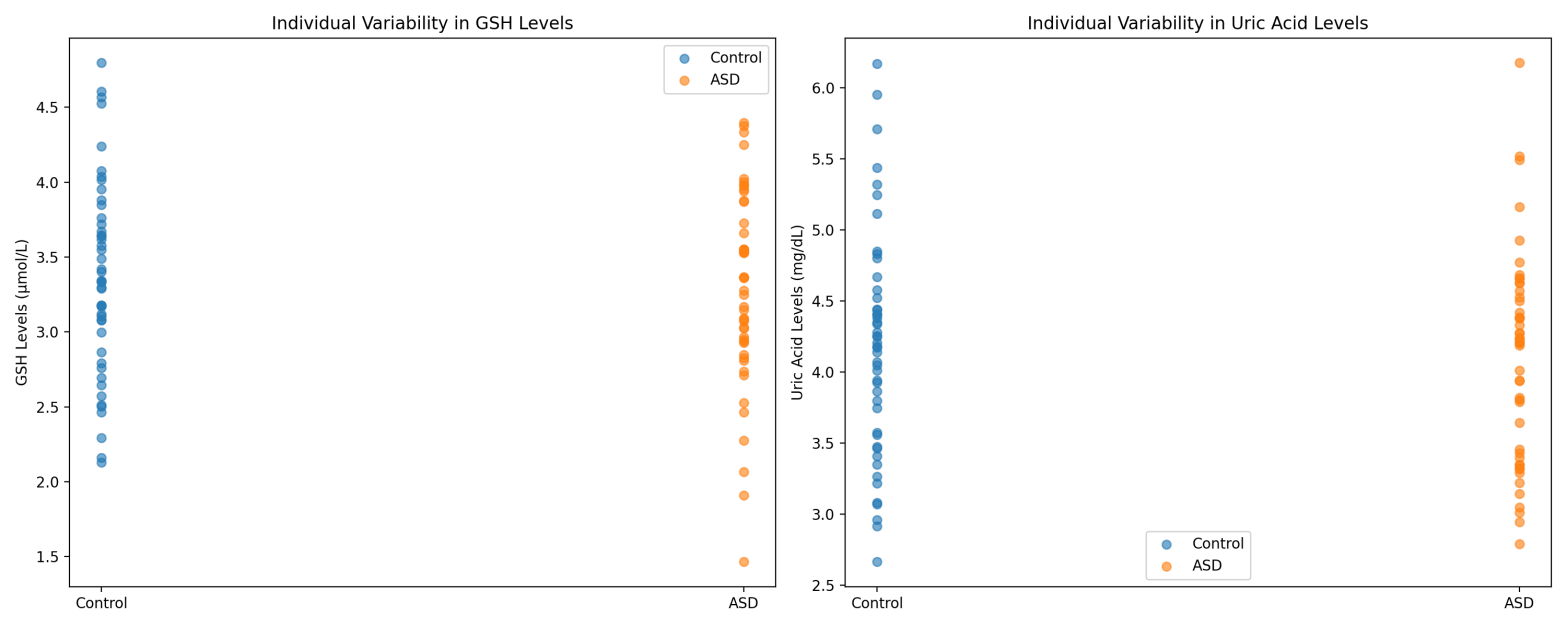
Individual variability in MDA levels, GSH and uric acid levels. MDA: malondialdehyde, GSH: glutathione.

Table 2 presents a comparative analysis of saliva oxidative stress biomarkers and gingival health status. The mean plaque index was significantly higher in the control group (0.42 ± 0.38) compared to the ASD group (0.18 ± 0.36), with a mean difference of 0.24 (95% CI: 0.08 to 0.40; p = 0.003). For oxidative biomarkers, the confidence intervals showed no significant mean differences between groups, indicating a range of variability within and across groups for MDA (95% CI: −20.3 to 53.4 μM/L), GSH (95% CI: −4.5 to 10.9 μM/L), and uric acid (95% CI: −1.2 to 4.6 mg/dL). These confidence intervals emphasize the preliminary nature of our findings due to sample size limitations (Figure 2).

**Table 2:** Difference in saliva oxidative stress biomarkers and gingival health status between cases and control.

Variables	Study group	N	Mean	Std. deviation	95% CI	p-value	t-test
Gingival index (scale)	Control	45	0.083	0.15		0.335	Non-significant
	Case	45	0.049	0.17			
Plaque index (scale)	Control	45	0.42	0.38	0.08–0.40	0.003	Significant
	Case	45	0.18	0.36			
Salivary malondialdehyde (μM/L)	Control	45	111.63	157.71	−20.3 to 53.4	0.141	Non-significant
	Case	45	175.32	239.71			
Salivary glutathione (μM/L)	Control	45	11.04	13.21	−4.5 to 10.9	0.178	Non-significant
	Case	45	16.27	22.22			
Uric acid (mg/dL)	Control	45	24.54	11.49	−1.2 to 4.6	0.320	Non-significant
	Case	45	27.36	15.02			

Table 3 presents the distribution of demographic characteristics across the control and case groups. Statistical analysis revealed no significant difference in gender distribution between the groups, with males constituting the majority in the control 27 (60%) and case 32 (71.1%) groups. Similarly, the residence variable did not show a significant difference, as most participants in both groups were from urban areas (40 (88.8%) participants in the control group and 43 (95.5%) participants in the case group). However, a significant difference was observed in the socioeconomic status; in the control group, 27 (60%) participants were from the low socioeconomic status, and 25 (55.6%) case group participants were classified under the medium socioeconomic status.

**Table 3:** Association between gender and study group.

Variable	Categories	Study group		p-value
		Control	Case	
Sex	Male	27 (60%)	32 (71.1%)	0.267
	Female	18 (40%)	13 (28.9%)	
Residence	Rural	5 (11.2%)	2 (4.5%)	0.432
	Urban	40 (88.8%)	43 (95.5%)	
Socioeconomic status	Low	27 (60%)	7 (15.5%)	0.045
	Medium	13 (28.8%)	25 (55.6%)	
	High	5 (11.2%)	13 (28.9%)	
Total		45 (100%)	45 (100%)	

## Discussion

The descriptive statistics for various parameters measured in the participants included age, gingival index, plaque index, salivary MDA, salivary GSH, and uric acid levels. The mean age of the participants was 8.38 ± 2.19 years. This age range is consistent with earlier studies investigating oral health and salivary biomarkers in children and adolescents [22]. The mean gingival index was 0.06 ± 0.166, indicating a remarkably low level of gingival inflammation in the study population. This result aligns with children's desired gingival health status, as the prevalence and severity of gingival diseases tend to increase with age [23,24].

The results of the present study showed a moderate level of plaque accumulation. This statement is consistent with earlier investigations that revealed higher plaque levels in children than adults, potentially owing to poor oral hygiene practices and dietary habits [25]. The mean salivary MDA level is a widely used oxidative stress biomarker, and different characteristics, including age, diet, and systemic conditions, can affect its levels. Other analyses have documented salivary MDA levels in children and adolescents, with some investigations showing higher levels in people with oral diseases or systemic conditions [26]. Salivary GSH is an essential antioxidant in saliva, and its levels can be affected by oxidative stress and inflammatory conditions. Uric acid is another salivary antioxidant affected by various factors, including diet, age, and systemic conditions, which can influence its levels [27].

The gender distribution agrees with several prior examinations documenting a higher prevalence of males in pediatric populations analyzed for oral health and salivary biomarkers. When the results are compared between control and case groups, there are significant differences, which is compatible with previous studies that have reported age-related variations in oral health and salivary biomarkers [28]. The control group exhibited a significantly higher mean plaque index than the case group, with a p-value of 0.003. The presence of plaque is a well-established risk factor for developing gingivitis and periodontitis, and various factors, including age, diet, and oral hygiene habits, can influence its levels. Behavioral factors, such as sensory processing issues in ASD, may impact oral hygiene, potentially leading to plaque buildup independent of oxidative stress mechanisms [24]. No substantial differences were marked between the control and case groups for gingival index, salivary MDA, salivary GSH, and uric acid levels. It is necessary to note that the lack of significant differences in these parameters may be affected by various factors [29]. The factors that may cause this result are variations in the host's immune response. The inflammatory response to plaque can vary between individuals due to differences in immune function, which may modify the clinical manifestations of gingivitis despite similar plaque levels. Plaque composition, in which specific microbial composition of the plaque biofilm can influence its pathogenicity and the resulting gingival inflammation. Additionally, the duration of plaque accumulation in gingivitis usually develops after a certain period. The cross-sectional nature of this study may have captured a time before elevated plaque levels in controls led to clinically detectable gingivitis [30].

The sex distribution agrees with several prior examinations documenting a higher prevalence of males in pediatric populations analyzed for oral health and salivary biomarkers. When the results were compared between the control and case groups, significant differences were observed, which is compatible with previous studies that reported age-related variations in oral health and salivary biomarkers [28]. The mean plaque index was significantly higher in the control group than in the case group (P = 0.003). The presence of plaque is a well-established risk factor for gingivitis and periodontitis, and various factors, including age, diet, and oral hygiene habits, can influence its levels [24]. No substantial differences were observed between the control and case groups regarding gingival index, salivary MDA, salivary GSH, and uric acid levels.

It should be noted that the lack of significant differences in these parameters may be affected by various factors [29]. The factors that may cause this result are variations in the host's immune response. The inflammatory response to plaque can vary between individuals due to differences in immune function, which may modify the clinical manifestations of gingivitis despite similar plaque levels. Plaque composition, in which the specific microbial composition of the plaque biofilm can influence its pathogenicity and resulting gingival inflammation. Additionally, plaque accumulation in gingivitis usually develops after a certain period. The cross-sectional nature of this study may have captured the time before elevated plaque levels in controls led to clinically detectable gingivitis [30].

The study’s focus on only two biomarkers and sample homogeneity are noted limitations. Furthermore, as a cross-sectional study, it only provides a temporal snapshot, limiting causal conclusions. Future studies should explore additional oxidative markers and examine the longitudinal impact of oxidative stress on ASD-related health.

## Conclusions

Contrary to the original hypothesis, none of the biomarkers showed statistically significant differences between the autism and control groups. However, compared to autistic participants, ordinarily developing children showed significantly more considerable dental biofilm accumulation according to objective plaque index values. Clinical gingival index examinations did not reveal intergroup differences; however, further long-term research is necessary. Limitations include the limited sample size and absence of confounders such as age, medication use, and socioeconomic status. From an oral health equality perspective, increasing family awareness of the heightened caries risk associated with neurotypical adolescents is worth considering, although the conclusions are preliminary. Expanded research with more resources could reveal new avenues for creating targeted preventative initiatives that benefit all.
